# Differential impact of South Korea’s 2019 E-cigarette advisory on adult tobacco product use: an interrupted time series analysis by product type and subgroup

**DOI:** 10.1186/s12889-026-27451-9

**Published:** 2026-04-24

**Authors:** Yuri Lee, Stephen O’Neill, Zia Sadique, Matt Egan

**Affiliations:** 1 Department of Public Health, Environments and Society, School for Public Health Research, London School of Hygiene & Tropical Medicine (LSHTM), London, UK; 2 Department of Health and Medical Information, Myongji College, Seoul, Republic of Korea; 3 Department of Health Services Research and Policy, Faculty of Public Health and Policy, London School of Hygiene & Tropical Medicine (LSHTM), London, UK

**Keywords:** Electronic cigarettes, Interrupted time-series, Tobacco policy, Public health advisory, Subgroup analysis, South Korea

## Abstract

**Background:**

In October 2019, South Korea’s Ministry of Health and Welfare issued a national advisory urging the public to refrain from using liquid-type/ electronic cigarettes (e-cigarettes) due to international concerns over vaping-related lung injuries. This study evaluates changes in e-cigarette use trends using nationally representative repeated cross-sectional survey data.

**Methods:**

We conducted an interrupted time-series (ITS) analysis using age-standardized prevalence data on current use of cigarettes, e-cigarettes (2013–2023), and heated tobacco products (HTPs, 2019–2023) from the Korea National Health and Nutrition Examination Survey. ITS regression models with Newey–West standard errors assessed pre-intervention trends, immediate level changes, and post-intervention trend differences. Subgroup analyses were performed by gender, age, region, and income. We also conducted sensitivity analyses using ± 1-year intervention timings and presented counterfactual projections.

**Results:**

E-cigarette use had been increasing before 2019 (+ 0.43% points/year, 95% CI: 0.11, 0.75), and a decline was observed in 2019 relative to prior trends (–1.33 pp, 95% CI: − 2.54, − 0.12), with no significant change in trend post-intervention. Subgroup effects varied, with men (–2.35 pp, 95% CI: − 4.39, − 0.30), middle-aged adults (e.g. 40–49 years: − 2.94 pp, 95% CI: − 5.36, − 0.52), and low-income individuals (–2.65 pp, 95% CI: − 3.07, − 2.24) showing the largest reductions. No compensatory increases were observed in cigarette or HTP use. Sensitivity tests confirmed the robustness of results to alternate intervention dates.

**Conclusions:**

The 2019 EVALI outbreak and the South Korean government’s advisory were associated with a decline in e-cigarette use at the population level, particularly among high-use subgroups. These findings suggest a potential role for timely public health advisories and risk communication in shaping behavior, even in the absence of binding regulations. The intervention illustrates how timely risk communication may contribute to measurable changes in health behavior at the population level.

**Supplementary Information:**

The online version contains supplementary material available at 10.1186/s12889-026-27451-9.

## Introduction

Tobacco use remains a leading cause of preventable disease and death globally. Despite steady declines in smoking prevalence in many countries, about 22.3% of the world’s adult population still used tobacco in 2020 [1], contributing to an enormous burden of cardiovascular, respiratory, and neoplastic diseases [2]. South Korea historically had one of the highest smoking rates among adult men, reaching 66.3% in 1998, but following strong tobacco control policies, male smoking prevalence fell to 30.0% by 2022, while female smoking remained low at 5.0% [3]. The gender and socioeconomic disparities in smoking, however, persist as critical public health issues [4, 5].

The nicotine product landscape in Korea has shifted over the past decade with the introduction of e-cigarettes in 2008 and heated tobacco products (HTPs) in 2017, both promoted as safer alternatives and rapidly adopted by younger users [6, 7]. This rapid uptake was driven in large part by deliberate industry strategies, including aggressive promotion of flavored products and the framing of these devices as harm-reduction tools [8, 9]. In the United States, e-cigarette use among high school students rose sharply from 7.9% in 2017 to 20.2% in 2019, prompting public health campaigns and regulatory actions to curb youth vaping [10]. At the same time, long-term health effects of vaping remained uncertain, and accumulating evidence began to suggest that e-cigarette aerosol can contain toxic metals and chemicals harmful to the lungs and cardiovascular system [11]. By February 25, 2020, concerns about e-cigarettes crystallized with the outbreak of e-cigarette or vaping product use-associated lung injury (EVALI) in the U.S., which hospitalized 2,807 people and resulted in 68 deaths nationwide, with investigations identifying vitamin E acetate—an additive in illicit cannabis vaping products—as a key causal agent [12, 13]. Although the EVALI epidemic was primarily linked to contaminated tetrahydrocannabinol (THC) vapes rather than nicotine e-cigarettes [10], its occurrence cast a spotlight on the potential dangers of vaping and led to swift policy responses in various countries.

South Korea was among the first countries outside the U.S. to respond decisively to the EVALI crisis. On October 23, 2019, the Ministry of Health and Welfare (MOHW) issued a strong public warning urging people to stop using liquid-type e-cigarettes, following the U.S. outbreak of vaping-related lung injury and a severe domestic case involving a 30-year-old user [14]. The broader regulatory context for e-cigarettes and heated tobacco products in South Korea is summarized in Supplementary Table S1. It represented an extraordinary intervention, essentially a recommendation to refrain from a legal consumer product without a formal ban. Heated tobacco products (HTPs) were not targeted by the 2019 advisory despite gaining market share. Given their rapid uptake, some raised concerns about potential substitution among discouraged e-cigarette users, though this study focuses on liquid-type e-cigarette trends [15].

While Korea’s e-cigarette advisory was a bold public health measure, its actual impact on population behavior was unknown. Evidence from other settings was limited. Some U.S. data suggested that e-cigarette sales and use declined temporarily during the EVALI scare in late 2019 [16, 17], and surveys documented increased risk perceptions about vaping among adults and changes in e-cigarette beliefs following extensive news coverage of the outbreak [10]. However, no prior study had formally evaluated the population-level effect of a governmental warning on e-cigarette use. South Korea’s situation offered a unique case study, with robust national surveillance data available to examine trends before and after the advisory. Importantly, understanding any collateral effects on other tobacco product use is vital for policy evaluation – a decline in vaping might lose public health benefit if offset by greater smoking or other product uptake.

We aimed to assess the population-level impact of the October 2019 e-cigarette use advisory in South Korea on subsequent trends in adult tobacco use. Using an interrupted time-series approach, we analyzed national survey data to quantify changes in prevalence of liquid e-cigarette use associated with the advisory, and to determine whether there were any concomitant changes in conventional cigarette smoking rates. We also analyzed trends in heated tobacco products used descriptively to check for evidence of product substitution. Additionally, subgroup analyses by sex, age, region, and income were conducted to identify any differential responses to the advisory across demographics. By evaluating this natural experiment, our study provides evidence on the effectiveness of a large-scale risk communication intervention in reducing harmful product use. The findings will inform ongoing tobacco control efforts in South Korea, which align with the World Health Organization (WHO) Framework Convention on Tobacco Control (FCTC) and Sustainable Development Goal (SDG) target 3.a (strengthening implementation of the WHO FCTC), and offer lessons for other countries facing the dual challenge of regulating novel tobacco products while continuing to reduce smoking prevalence.

## Methods

### Data source and study population

We utilized aggregated data from the Korea National Health and Nutrition Examination Survey (KNHANES), a nationally representative annual survey of the Korean population’s health behaviors and outcomes. Specifically, we extracted yearly prevalence estimates of current tobacco product use among adults aged 19 years and older for the period 2007 through 2023. KNHANES is a repeated cross-sectional survey conducted by the Korea Disease Control and Prevention Agency, employing a complex stratified multistage sampling design to provide nationally representative estimates [3]. The survey is conducted annually with data collected continuously throughout each calendar year, and official summary statistics are released annually through the Korean Statistical Information Service (KOSIS). In KNHANES, current tobacco product use is assessed through standardized self-reported questionnaire items asking respondents whether they currently use each tobacco product type, typically defined as use within the past 30 days at the time of survey administration. All data were obtained in aggregated and anonymized from through the Korean Statistical Information Service (KOSIS) online portal, which publishes official KNHANES results. Because we used only publicly available, de-identified summary data, this study was not subject to institutional review board approval.

We examined three tobacco outcomes: (1) Conventional cigarette smoking (manufactured combustible cigarettes), (2) Liquid-type e-cigarette use (nicotine vaping devices using liquid e-liquids), and (3) Heated tobacco product (HTP) use (devices like IQOS that heat tobacco sticks). In KNHANES, current use was defined as using the product at least once in the past month at the time of survey. For each year, we used the age-standardized prevalence (% of adults) as reported by KNHANES. Age standardization was done by KNHANES statisticians to adjust for any demographic shifts over time. We analyzed age-standardized rates to allow fair comparisons of prevalence across the years.

Data for cigarette smoking was available from 2007 to 2023. E-cigarette use was first surveyed in 2013, so data are available from that year onward. Heated tobacco product (HTP) use was first captured in 2019 KNHANES, one year after these products entered the Korean market [18]. Accordingly, the main analysis for liquid-type e-cigarettes covers the period from 2013 to 2023, when prevalence became measurable, while the cigarette analysis spans 2007 to 2023. Because no HTP data existed before the intervention year and only a limited number of post-intervention years are available, HTPs were not included in the formal interrupted time-series regression. Instead, their trends were described using simple descriptive methods. Because the prevalence of heated tobacco product use remained relatively stable over the period for which data were available (Figure S1), the potential risk of bias due to product substitution in the interrupted time-series analysis is likely to be limited.

### Interrupted time-series analysis

We applied an interrupted time-series (ITS) design to evaluate the impact of the October 2019 advisory on e-cigarette and cigarette use prevalence. ITS is a strong quasi-experimental approach for assessing longitudinal effects of population-level interventions when randomized trials are not feasible [19]. In our ITS regression models, the intervention was defined as occurring at the end of 2019, corresponding to the advisory issuance in October 2019. Given that our data are annual, we treated 2019 as the intervention time point (with 2013–2018 as the pre-intervention period for e-cigarettes, and 2007–2018 for cigarettes).

Prior to model specification, we visually inspected the time-series trends and conducted exploratory regression diagnostics to assess the plausibility of a linear trend during the pre-intervention period. For conventional cigarettes, the decline from 2007 to 2018 followed an approximately linear trajectory. For e-cigarettes, although earlier years showed fluctuations associated with market diffusion, the overall trend between 2013 and 2018 was reasonably approximated by a linear increase. Given the limited number of annual observations, a linear specification was considered the most parsimonious and interpretable model for the baseline trend. Each outcome was modeled as:

$$\:{Y}_{t}={\beta\:}_{0}+{\beta\:}_{1}\times\:Tim{e}_{t}+{\beta\:}_{2}\times\:Interventio{n}_{t}+{\beta\:}_{3}\times\:Time\:Afte{r}_{t}+{\epsilon}_{t},$$

where $\:{Y}_{t}$ is the outcome prevalence (in percentage points) in year *t*; Time is a continuous variable indicating time since start of series; Intervention is a dummy variable (0 before 2019, 1 for 2019 and after) representing the level change at the intervention; Time After is a continuous variable counting years after 2019 (0 before 2019, 1 for 2020, 2 for 2021, etc.) representing the change in slope after intervention; and $\:{\epsilon}_{t}$​ is the error term. From this model, $\:{\beta\:}_{0}$​ estimates the baseline level (intercept) at the start of the series, $\:{\beta\:}_{1}$​ the baseline trend (annual change) prior to intervention, $\:{\beta\:}_{2}$​ the immediate change in prevalence in the intervention year (i.e., the difference between observed 2019 level and the counterfactual predicted 2019 level based on prior trend), and $\:{\beta\:}_{3}$​ the change in the post-intervention trend relative to the pre-intervention trend. We report these coefficients in percentage points (pp) along with 95% confidence intervals (CI). The models were fitted by ordinary least squares regression. To account for potential autocorrelation in the annual time-series data (e.g., serial correlation of unobserved influences from year to year), we utilized Newey–West standard errors with a lag of 1 year. The Newey–West estimator is a heteroskedasticity and autocorrelation-consistent covariance estimator commonly used in time-series regression, which adjusts the standard errors to yield valid inference even when errors are autocorrelated up to the specified lag.

Separate ITS analyses were conducted for (a) cigarette smoking prevalence (2007–2023) and (b) e-cigarette use prevalence (2013–2023). HTPs were not modeled due to the limited availability of data, with only five post-2019 data points. However, we plotted HTP prevalence over time to qualitatively assess any noticeable change over time.

We stratified the e-cigarette ITS analysis by key demographic subgroups to explore heterogeneous effects of the advisory. Specifically, we created separate annual series (2013–2023) for subpopulations by sex (male vs. female), by age group (19–29, 30–39, 40–49, 50–59, 60–69, ≥ 70 years), by residential region (urban vs. rural, per KNHANES definitions), and by household income level. KNHANES reports smoking prevalence by quintiles of equivalized household income (from “low” for the bottom 20% up to “high” for the top 20%). ITS analyses analogous to the main model were then fit for each subgroup’s e-cigarette prevalence trend. Because some subgroup sample sizes (e.g., older age groups) were smaller, these estimates had wider variability; we focus interpretation on the magnitude and confidence interval of the intervention effect in each subgroup rather than formal interaction tests. Annual sample sizes used in the ITS are provided in Supplementary Table S2.

To aid interpretation of effect size, we projected a counterfactual trend for e-cigarette use prevalence in the absence of the EVALI outbreak and subsequent advisory. This was done by extending the pre-2019 linear trend ($\:{\beta\:}_{1}$​) forward for 2019–2023, using the model without the intervention term. The projected values and their 95% CI were then compared against the observed post-2019 prevalence to visualize differences attributable to the intervention. In graphs, we indicate the advisory timing with a vertical dashed line and the counterfactual trajectory with a dotted line.

We conducted two sensitivity analyses to check the robustness of our findings to the choice of intervention date. First, we performed a placebo test by assuming an intervention start date one year earlier (2018) – essentially asking whether a significant effect would have been detected had we incorrectly assumed an intervention before the actual advisory. A lack of any effect in 2018 would support the validity of the 2019 impact. Second, we tested an alternative specification with the intervention in 2020, to assess if any delayed or residual impact might appear if the true behavioral change occurred slightly later for instance, if some users only quit after observing more news or policy action in early 2020. For both scenarios, we refit the ITS regression for e-cigarette prevalence with the dummy and trend break at 2018 and at 2020, respectively, and examine the coefficients for level and slope changes. Additionally, we qualitatively examined 2020 data in light of the COVID-19 pandemic, which began that year, as a potential confounder, although pandemic effects on smoking and vaping in Korea were not clearly directional a priori.

All analyses were conducted using Stata 18 SE. Statistical significance was interpreted based on 95% CI exclusion of zero rather than *p*-values, in line with recent recommendations to focus on effect sizes and uncertainty.

## Results

### Pre- and post-2019 tobacco use prevalence

Table 1 summarizes the prevalence of tobacco product use in the periods before (2013–2018) and after (2019–2023) the October 2019 advisory. During this time, adult cigarette smoking in Korea declined markedly, with mean age-standardized prevalence falling from 23.3% to 19.7%—a relative decrease of approximately 15.5%. Liquid-type e-cigarette use, by contrast, rose slightly in crude prevalence (from 2.5% to 2.7%) and more notably in age-standardized prevalence (from 2.8% to 4.5%). Although still far lower than cigarette smoking, e-cigarette use became more prevalent in the latter period. Heated tobacco products (HTPs), which were virtually absent before 2017, reached an average age-standardized prevalence of 5.6% in 2019–2023, exceeding e-cigarettes in population uptake during the same period (Table 1).

**Table 1:** Pre–Post 2019 Characteristics of Adult Tobacco Product Use in Korea

Product type	Period	Crude prevalence (%)	Age-standardized prevalence (%)
Conventional cigarettes	2013–2018	22.2	23.3
	2019–2023	18.6	19.7
Liquid-type e-cigarettes	2013–2018	2.5	2.8
	2019–2023	2.7	4.5
Heated tobacco products	2019–2023	4.5	5.6

*Caption*: Values represent mean crude and age-standardized prevalence (%) of current tobacco product use among adults aged ≥ 19 years in the pre-intervention period (2013–2018) and post-intervention period (2019–2023). Age-standardized prevalence estimates were obtained from the Korea National Health and Nutrition Examination Survey (KNHANES) and accessed via the Korean Statistical Information Service (KOSIS). Heated tobacco products were analyzed descriptively for 2019–2023 due to data availability. Prevalence values are simple averages of annual estimates within each period

**Fig. 1:**
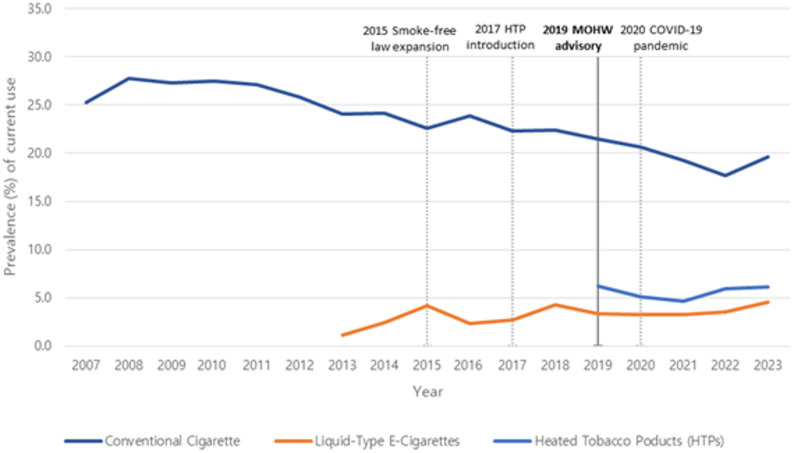
Trends in Cigarette and E-Cigarette Use Among Adults in Korea, 2007–2023. Caption: Trends in current use (%) of three tobacco product types among adults aged ≥ 19 years in the Republic of Korea. The prevalence of conventional cigarette smoking showed a gradual decline from 2007 to 2021, followed by a slight increase in 2023. Liquid-type e-cigarette use emerged after 2013, peaking around 2015, while heated tobacco product (HTP) use, first introduced in 2017, demonstrated a steady upward trend through 2023. Data are age-standardized estimates obtained from the Korea National Health and Nutrition Examination Survey (KNHANES) and accessed via the Korean Statistical Information Service (KOSIS). Vertical dashed lines indicate key policy and contextual events potentially influencing tobacco use trends in South Korea. The solid vertical line highlights the October 2019 Ministry of Health and Welfare advisory on liquid-type e-cigarettes, the primary intervention examined in the interrupted time-series analysis

Figure 1 illustrates the time trends in prevalence of each product category from 2007 to 2023. Cigarette smoking shows a steady downward trajectory over the entire period. The smoking rate among adults fell from about 25.3% in 2007 to 19.3% by 2021, with a slight uptick to 19.6% in 2023. The long-term decline in smoking is attributed to intensified tobacco control policies such as tax hikes, smoke-free laws, graphic warnings, etc. and changing social norms in Korea [20]. Liquid e-cigarette use emerged in the data from 2013 onward, rising sharply to a peak around 2015 and then declining. Specifically, liquid-type e-cigarette use rose sharply from 1.1% in 2013 to 4.2% in 2015, then declined to 2.3% in 2016 and remained between 2.7% and 4.3% from 2017 to 2019. This mid-decade spike corresponds to the initial boom of vaping in Korea, followed by a downturn likely driven by negative publicity about unregulated vaping liquids and early regulatory actions such as the inclusion of e-cigarettes in indoor smoking bans implemented in 2015 [21]. After 2018, e-cigarette use was relatively flat or slightly increasing until 2020. Heated tobacco product use starts near zero prior to 2017, then climbs continuously through 2023. HTP prevalence reached roughly 6.1% by 2023, reflecting rapid adoption of products like IQOS and Lil in the Korean market [22, 23]. It is noteworthy that by the end of the study period, HTPs had become a significant component of tobacco use in Korea, rivaling conventional smoking rates among men. Visual inspection of Fig. 1 suggests a potential impact in 2019 for e-cigarettes, as the orange line shows a visible dip between 2019 and 2020. After this point, e-cigarette prevalence remains lower than the peak levels seen in the mid-2010s, despite some rebound between 2021 and 2023. In contrast, no clear disruption is observed in the smoking trend in 2019, as the blue line continues its smooth downward trajectory (Fig. 1).

### Impact of the 2019 advisory: main interrupted time-series results

**Fig. 2:**
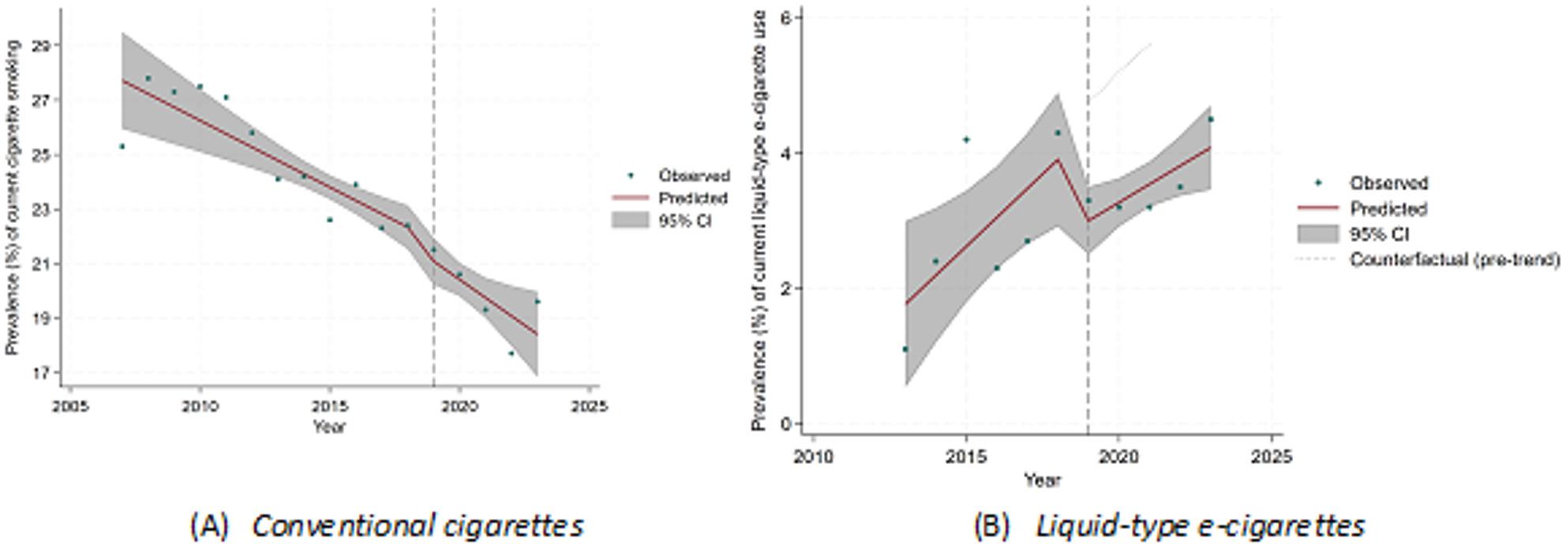
Interrupted Time-Series Analysis of Adult Smoking Trends in South Korea (2007–2023). Note. Vertical dashed lines indicate the October 2019 intervention point corresponding to the Ministry of Health and Welfare’s national advisory to refrain from using liquid-type e-cigarettes. Solid lines represent fitted ITS regression estimates from interrupted time-series models, shaded areas indicate 95% confidence intervals, and dots denote observed annual prevalence (%) among adults aged ≥ 19 years. In panel (**B**), the dotted line represents the counterfactual projection based on the pre-intervention trend, illustrating the expected trajectory in the absence of the 2019 advisory. **A** Conventional cigarettes. The fitted ITS regression indicates a sustained long-term decline in adult cigarette smoking from 2007 to 2023. Confidence intervals are relatively narrow, and no abrupt level change is evident following the 2019 intervention, suggesting continuation of a pre-existing structural downward trend. **B** Liquid-type e-cigarettes. Pre-intervention years (2013–2018) show an increasing trend in use, followed by an estimated immediate decline around the October 2019 advisory and a modest rebound thereafter. However, the 95% confidence intervals are wide, indicating substantial uncertainty around the magnitude of post-intervention effects, and a null effect cannot be ruled out when compared with the counterfactual projection

Table 2 presents the ITS regression estimates for adult prevalence of conventional cigarette smoking and liquid e-cigarette use, evaluating changes associated with the October 2019 advisory. For cigarette smoking, the regression confirms a highly significant downward pre-intervention trend of − 0.49% points per year (95% CI: − 0.71, − 0.27) from 2007 up to 2018. The immediate level change in 2019 for cigarettes was estimated at − 0.76 pp (95% CI: − 2.06, 0.54). Although a negative value, this change was not statistically distinguishable from zero (the CI includes zero), indicating no clear evidence that EVALI and the associated advisory produced an abrupt shift in smoking prevalence. Furthermore, there was no significant change in trend after 2019 (β₃ = − 0.18 pp/year, 95% CI: − 0.74, 0.38). The post-2019 slope was slightly more negative than before, but the difference was not meaningful relative to the variability (Table 2). Figure 2A displays these results graphically. The fitted ITS regression line for cigarettes follows a smooth downward path through 2019 and beyond, with no evident slope break. The 95% confidence band around the trend is narrow and closely aligned with the projected continuation of the pre-2019 trend. The counterfactual and actual trends for smoking are nearly identical (Fig. 2).

**Table 2:** Interrupted Time-Series (ITS) Regression Results by Tobacco Product Type among Adults, South Korea, 2007–2023

Product Type	Baseline level, β₀ (pp)	Pre-intervention trend, β₁ (pp/year)	Immediate level change in 2019, β₂ (pp)	Change in post-intervention trend, β₃ (pp/year)
Conventional Cigarettes	27.72 (95% CI: 25.94, 29.50)	–0.49 (95% CI: − 0.71, − 0.27)	–0.76 (95% CI: − 2.06, 0.54)	–0.18 (95% CI: − 0.74, 0.38)
Liquid-Type E-Cigarettes	1.76 (95% CI: 0.53, 2.99)	+ 0.43 (95% CI: 0.11, 0.75)	–1.33 (95% CI: − 2.54, − 0.12)	–0.16 (95% CI: − 0.58, 0.26)

Estimates are from ITS regression models fitted to aggregated annual prevalence data with Newey–West standard errors (lag 1). All coefficients are reported in percentage points (pp) with 95% confidence intervals; β₁ and β₃ are expressed as pp per year. β₀ represents the estimated baseline prevalence level, β₁ the annual pre-intervention trend, β₂ the immediate level change following the October 2019 Ministry of Health and Welfare (MOHW) advisory, and β₃ the change in trend after the intervention. Heated tobacco products were not included in the ITS regression table due to data availability only from 2019 onward; their trends are described descriptively in the Results

In contrast, the ITS regression for liquid-type e-cigarette use shows a significant change in level in 2019 relative to prior trends. Prior to 2019 (2013–2018), e-cigarette use was trending upward at + 0.43% points per year (95% CI: +0.11, + 0.75). We estimate a − 1.33 pp drop in e-cigarette prevalence in 2019 (95% CI: − 2.54, − 0.12), coinciding with the period of the EVALI outbreak and the Korean government advisory. This indicates that the observed 2019 e-cigarette use rate was over a full percentage point lower than expected based on prior trends. Given that e-cigarette prevalence in 2018 was 4.3%, a 1.33 pp decline reflects a relative reduction of approximately 31%. After 2019, the trend in e-cigarette use flattened. The post-2019 trend was an estimated − 0.16 pp per year, which is 0.59 pp/year lower than the pre-trend, although this change in slope did not reach statistical significance (95% CI for slope change: − 0.58, + 0.26). Figure 2B illustrates this pattern clearly, showing a downward shift in the fitted line at the 2019 mark and a nearly horizontal trajectory afterward (Table 2). The counterfactual projection extends the pre-2019 growth and lies above the observed values each year post-intervention. By 2023, the counterfactual e-cigarette prevalence is about 5.0% whereas the actual observed prevalence is 2.8%, with the observed values remaining below the projected counterfactual trend. However, the shaded confidence region for the projection and actual overlap substantially, reflecting uncertainty – it’s possible that e-cigarette use might have plateaued or declined even without the advisory, given market changes and the emergence of HTPs (Fig. 2).

There was a decline in e-cigarette use without a corresponding increase in smoking prevalence. Additionally, the post-2019 trend remained relatively flat compared to the pre-2019 increase, although the change in slope was not statistically significant.

### Subgroup analyses of E-cigarette use

**Fig. 3:**
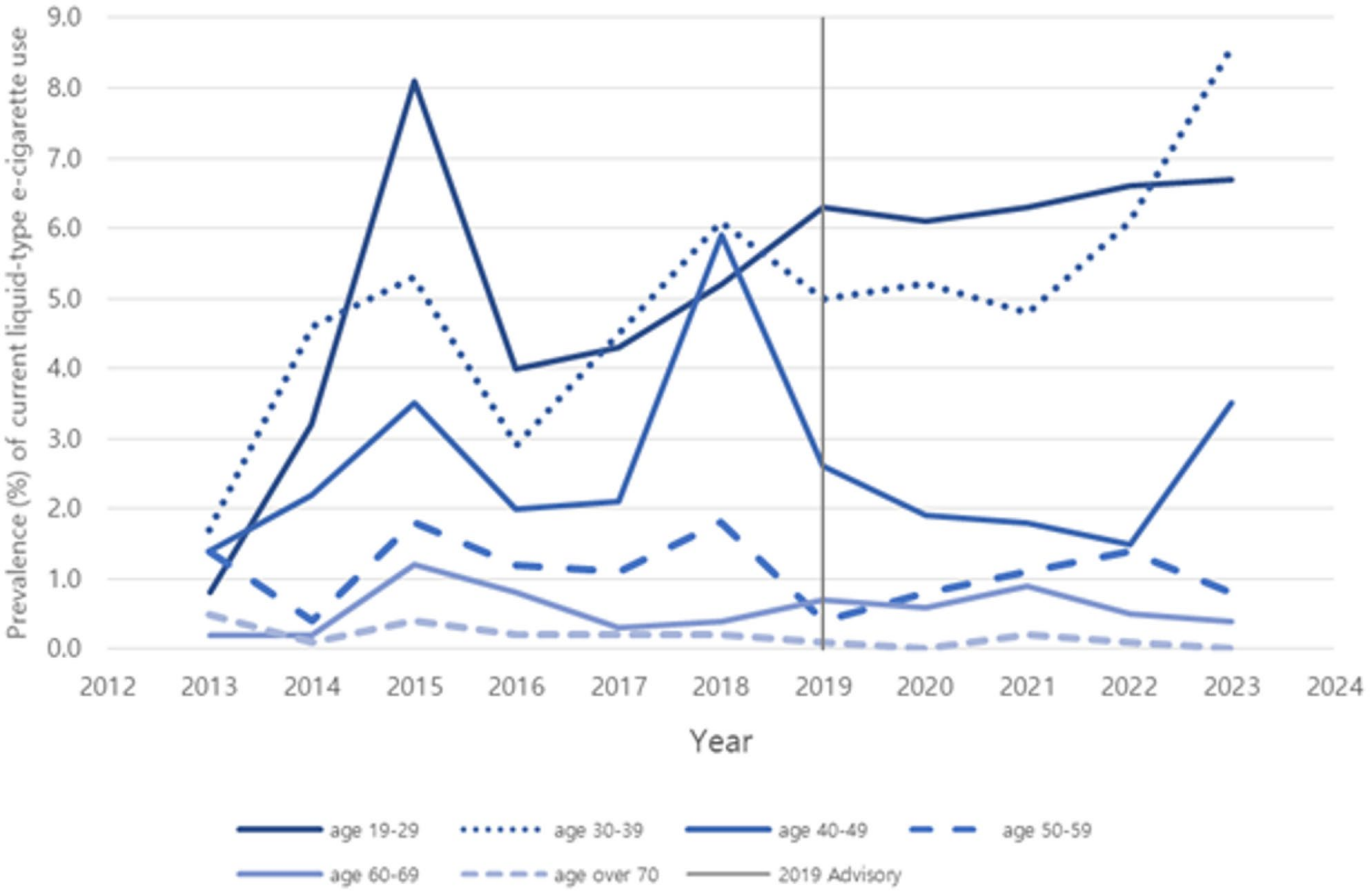
Trends in Liquid-Type E-Cigarette Use by Age Group, 2013–2023. Caption: Trends in current liquid-type e-cigarettes use among adults aged ≥ 19 years in Korea, in the Republic of Korea, stratified by age group. Current use is defined as self-reported use of liquid-type e-cigarettes within the past month at the time of the survey, and therefore year-to-year variation is expected. Prevalence was consistently highest among younger adults aged 19–29 years, with a pronounced spike in 2015, followed by a decline and subsequent stabilization after 2018. Adults aged 30–39 years showed a gradual increase after 2020, reaching the highest level in 2023. In contrast, prevalence among adults aged 50 years and older remained consistently low throughout the study period. The vertical dashed line indicates the timing of the October 2019 Ministry of Health and Welfare advisory on liquid-type e-cigarettes. Data are age-standardized estimates obtained from the Korea National Health and Nutrition Examination Survey (KNHANES), 2013–2023

Figure 2 illustrates trends in liquid-type e-cigarette use by age groups from 2013 to 2023. Prevalence was consistently highest among adults aged 19–29 years, peaking around 2015 and declining thereafter. Adults aged 30–39 showed a gradual increase after 2020, reaching their highest level of use in 2023. In contrast, e-cigarette use among older adults, especially those aged 50 and above, remained low and relatively stable throughout the study period (Fig. 3). These patterns show variation across age groups around 2019, which we further evaluate using subgroup-specific interrupted time-series models (Table 3).

**Table 3:** Interrupted Time-Series (ITS) Regression Results for Liquid-Type E-Cigarette Use by Subgroup, South Korea (2013–2023)

Category	Subgroup	Pre-intervention trend, β₁ (pp/year, 95% CI)	Immediate level change in 2019, β₂ (pp, 95% CI)	Change in post-intervention trend, β₃ (pp/year, 95% CI)
Gender	Male	+ 0.65 (0.08, 1.21)	–2.35 (–4.39, − 0.30)	–0.27 (–0.92, 0.39)
	Female	+ 0.13 (0.07, 0.20)	–0.05 (–0.49, 0.39)	–0.01 (–0.20, 0.17)
Age Group	19–29 yrs	+ 0.61 (–0.23, 1.44)	–0.25 (–2.80, 2.31)	–0.48 (–1.31, 0.36)
	30–39 yrs	+ 0.55 (0.09, 1.01)	–1.79 (–3.61, 0.03)	+ 0.26 (–0.52, 1.03)
	40–49 yrs	+ 0.59 (0.06, 1.12)	–2.94 (–5.36, − 0.52)	–0.45 (–1.29, 0.38)
	50–59 yrs	+ 0.10 (–0.06, 0.26)	–1.01 (–1.78, − 0.24)	+ 0.04 (–0.19, 0.27)
	60–69 yrs	+ 0.03 (–0.13, 0.18)	+ 0.15 (–0.45, 0.75)	–0.10 (–0.27, 0.08)
	≥ 70 yrs	–0.04 (–0.09, 0.01)	–0.03 (–0.17, 0.12)	+ 0.03 (–0.03, 0.09)
Region	Urban residents	+ 0.43 (0.10, 0.77)	–1.30 (–2.58, − 0.02)	–0.18 (–0.61, 0.24)
	Rural residents	+ 0.28 (–0.10, 0.65)	–0.97 (–2.57, 0.63)	–0.04 (–0.46, 0.39)
Income Level	High income	+ 0.20 (–0.10, 0.49)	+ 0.04 (–1.43, 1.51)	–0.25 (–0.81, 0.32)
	Upper-middle income	+ 0.41 (0.05, 0.78)	–1.26 (–2.68, 0.16)	+ 0.22 (–0.20, 0.63)
	Middle income	+ 0.29 (–0.30, 0.88)	–1.93 (–4.07, 0.20)	+ 0.26 (–0.47, 0.99)
	Lower-middle income	+ 0.59 (0.25, 0.94)	–0.64 (–2.05, 0.77)	–0.88 (–1.38, − 0.38)
	Low income	+ 0.62 (0.56, 0.68)	–2.65 (–3.07, − 2.24)	–0.21 (–0.48, 0.06)

Estimates are from ITS regression models fitted to aggregated annual prevalence data with Newey–West standard errors (lag 1). All coefficients are reported in percentage points (pp) with 95% confidence intervals; β₁ and β₃ are expressed as pp per year. Interpretations are based on confidence intervals

Male adults were the primary users of e-cigarettes in Korea – for instance, in 2018, the prevalence of current e-cigarette use was 7.1% among men versus 1.1% among women. Correspondingly, the advisory’s effect was largely concentrated among men. Among males, e-cigarette use had been increasing before 2019 (β₁ = +0.65 pp/year, 95% CI: +0.08, + 1.21) and showed a significant immediate drop of − 2.35 pp (95% CI: − 4.39, − 0.30) in 2019. In contrast, females showed a very small baseline trend (+ 0.13 pp/year) and essentially no level change at 2019 (+ 0.05 pp, 95% CI: − 0.49, + 0.39). This sex difference is not surprising given that only ~ 1–2% of women vaped even at the peak; the female e-cigarette prevalence trend was nearly flat both before and after 2019. For men, however, the − 2.35 pp drop represents a sizable proportional decrease (male vaping prevalence declined from 7.1% in 2018 to 5.2% in 2020 per KNHANES). The post-trend for men was slightly downward (–0.27 pp/year) but not significant, suggesting the main effect was the immediate reduction without a further accelerating decline.

In the ITS models, adults aged 19–29 had a positive but non-significant pre-trend of + 0.61% points per year (95% CI: − 0.23, 1.44), and an estimated immediate level change of − 0.25 pp in 2019 (95% CI: − 2.80, 2.31). The lack of statistical significance suggests the advisory did not produce a measurable shift for this group, potentially because their vaping rates had already declined from earlier peaks. Among adults aged 30–39, the pre-intervention trend was significantly positive at + 0.55 pp/year (95% CI: 0.09, 1.01), with a borderline-significant immediate drop of − 1.79 pp in 2019 (95% CI: − 3.61, 0.03). Although the confidence interval includes zero, the point estimate indicates a substantial reduction, and the result should be interpreted cautiously given the associated uncertainty. Similarly, adults aged 40–49 exhibited one of the strongest responses: a significant − 2.94 pp level change (95% CI: − 5.36, − 0.52) and a positive pre-trend (+ 0.59 pp/year, 95% CI: 0.06, 1.12) that flattened slightly post-advisory. For the 50–59 age group, there was a modest pre-trend (+ 0.10 pp/year, 95% CI: − 0.06, 0.26) and a significant immediate drop of − 1.01 pp (95% CI: − 1.78, − 0.24). Older adults aged 60–69 and ≥ 70 showed minimal changes, with confidence intervals that included zero for both level and trend changes, suggesting no detectable effect. Overall, the strongest intervention-associated declines were observed among adults in their 30s to 50s. These groups likely comprised a large portion of the vaping population in 2019 and may have been most responsive to the health advisory. In contrast, younger adults may have already reduced use by that point, and older adults showed low baseline prevalence, leaving less room for change.

With respect to the region, we found that urban residents experienced a significant decline in e-cigarette use post-advisory (level change − 1.30 pp, 95% CI: − 2.58, − 0.02), while rural residents did not (–0.97 pp, 95% CI: − 2.57, + 0.63). Pre-intervention trends were positive in both regions, though only significant for urban areas. This suggests the advisory’s effect on vaping was concentrated in cities, which is plausible given higher initial prevalence and greater media reach/health campaign penetration in urban settings. Rural e-cigarette use was relatively low and changes there were not statistically significant.

Stratifying by income quintile revealed a striking gradient in response. The lowest income group saw the most dramatic drop in e-cigarette use associated with 2019: − 2.65 pp (95% CI: − 3.07, − 2.24), with a clearly significant negative change. This group also had a notable pre-2019 upward trend (+ 0.62 pp/year) that essentially halted after the advisory. In contrast, the lower-middle income group showed no statistically significant immediate level change (–0.64 pp), but exhibited a significant post-2019 trend turned (–0.88 pp/year, 95% CI: − 1.38, − 0.38). This pattern may reflect a more gradual reduction in use over time; however, because post-intervention trend changes may also be influenced by contemporaneous factors unrelated to the advisory, this finding should be interpreted cautiously. The middle and upper-middle income categories had intermediate responses (point estimates around − 1.3 to − 1.9 pp for level change) but confidence intervals included zero, so evidence of an effect is weaker. Notably, the highest income group showed essentially no impact: a tiny + 0.04 pp level change (95% CI: − 1.43, + 1.51) and no trend difference. High-income individuals in Korea were somewhat less likely to use e-cigarettes initially. Our findings suggest that lower-income e-cigarette users were more likely to quit or reduce use following the advisory, whereas higher-income users largely maintained their usage rates. This could reflect differences in risk perception or financial flexibility – lower-income users may have been more risk-averse or deterred by the possibility of health costs. It is also possible that some higher-income vapers switched to other products like HTPs, which are relatively expensive rather than quitting nicotine entirely, a behavior not directly captured without individual tracking (Table 3).

### Trends in heated tobacco product use and potential substitution

Although HTPs were not subject to the intervention and we lacked pre-2019 data to formally model, we analyzed their trends to assess if former vapers might have switched to HTPs. Figure 1 showed a steady increase in HTP use from 2017 to 2023, with no obvious inflection around 2019. Figure S1 shows HTP prevalence by sex from 2019 to 2023. Among men, HTP use peaked at 10.3% in 2019, declined to 7.3% by 2021, and then modestly rebounded to 9.1% in 2023. Female use gradually increased from 1.9% in 2019 to 2.9% in 2023. These trends suggest no abrupt post-advisory shift, but rather a continuation of gradual changes in HTP use patterns. These data provide no indication that the 2019 e-cigarette advisory caused a sharp increase in HTP uptake, rather HTP adoption appears to have been driven by its own marketing and acceptance trajectory. Additionally, a majority of HTP users in Korea were dual or triple users (also smoking or vaping), and the introduction of HTPs did not significantly improve smoking cessation rates [18]. Thus, substitution of e-cigarettes with HTPs, if it occurred, was only one component of complex multi-product use behavior. Our results suggest that any such substitution was not large enough to be visible as a deviation in population-level HTP trends after the advisory.

### Sensitivity analyses

Our placebo and alternative timing analyses reinforced the inference that 2019 was the key inflection point for e-cigarette use. When we re-assigned the intervention to 2018, the ITS regression found no significant level change in 2018 (+ 0.082 pp, 95% CI: − 2.03, + 2.20) and no significant trend change (β₃ = − 0.264 pp/year, 95% CI: − 0.814, + 0.286). In other words, had we assumed a policy impact one year earlier (when in reality nothing specific occurred on a national scale), the model would not have detected any meaningful shift – lending credibility to the notion that the real changes observed in late 2019 were not merely part of a pre-existing fluctuation or secular trend. Conversely, using 2020 as the intervention year still yielded an immediate drop (–1.201 pp, 95% CI: − 2.379, − 0.024). This likely reflects the fact that by 2020, e-cigarette prevalence was indeed lower than the extrapolated pre-2019 trajectory, whether we attribute it to late 2019 or early 2020. The trend change in the 2020-intervention model was a small positive (+ 0.102 pp/year, CI: − 0.228, + 0.432), suggesting a slight rebound or leveling after the drop (Table 4). Importantly, the 2018 placebo test was null while the 2019 and 2020 tests showed consistent declines, supporting the interpretation that the observed decline occurred around late 2019. We also considered the potential influence of the COVID-19 pandemic in 2020: social distancing and respiratory health concerns could have conceivably impacted smoking/vaping behaviors. However, smoking in Korea continued to decline in 2020 and 2021 without an anomalous change, and e-cigarette use did not resurge, so the pandemic likely did not confound our main findings in a major way. If anything, heightened respiratory risk awareness during COVID-19 might have further discouraged vaping, complementing the advisory’s effect. The sensitivity analyses strengthen confidence in attributing the observed drop in e-cigarette use to the 2019 advisory (and its aftermath), rather than to unrelated background trends.

**Table 4:** Sensitivity Analyses Using Alternative Intervention Years (Liquid-type E-cigarettes), 2013–2023

Alternative intervention year	β₂ Level change (pp, 95% CI)	β₃ Trend change (pp/year, 95% CI)
2018 (placebo)	+ 0.082 (–2.032, 2.197)	–0.264 (–0.814, 0.286)
2019 (main)	–1.333 (–2.542, − 0.125)	–0.159 (–0.578, 0.261)
2020 (alternative)	–1.201 (–2.379, − 0.024)	+ 0.102 (–0.228, 0.432)

Estimates are from ITS regression models fitted to aggregated annual prevalence data with Newey–West standard errors (lag 1). All coefficients are reported in percentage points (pp) with 95% confidence intervals; β₃ is expressed as pp per year

## Discussion

This study provides the first comprehensive evaluation of a nationwide warning against e-cigarette use on actual usage patterns in the population. We found that the 2019 EVALI outbreak and the South Korean government’s advisory urging the public to stop using liquid e-cigarettes were associated with a decline in adult e-cigarette prevalence. After several years of growth, the prevalence in 2020 was approximately 1–2% points lower than expected based on preintervention trends. Notably, this reduction did not come at the expense of reversing the downward trend in cigarette smoking. Smoking prevalence continued to decrease at a similar rate, indicating no clear evidence that EVALI and the associated advisory produced an abrupt shift in smoking prevalence. This pattern may reflect longer-term declines in smoking driven by broader tobacco control policies rather than a distinct shift around 2019. However, because our analysis focuses on smoking prevalence rather than smoking intensity, we cannot exclude the possibility that some individuals increased cigarette consumption after discontinuing e-cigarette use. We also found no evidence of a substantial shift toward HTPs. HTP use had already been increasing since 2017 and continued along a similar trajectory during 2019–2020, suggesting that the decline in vaping may reflect risk avoidance rather than large-scale substitution.

Our subgroup analyses shed light on who was most affected by the advisory. The decline in e-cigarette use was driven primarily by men, who constitute the majority of vapers in Korea. Men responded strongly to the warning, while women showed essentially no change, aligning with gender norms in tobacco use. In terms of age, middle-aged adults exhibited the clearest drops in vaping prevalence. Younger adults, despite having the highest propensity to vape historically, did not demonstrate a statistically significant drop around 2019. Meanwhile, older adults had minimal e-cigarette usage and effect sizes were small with wide confidence intervals. An intriguing finding was the income differential in response: lower-income individuals saw the most pronounced reduction in vaping after the advisory, whereas higher-income individuals were relatively unaffected, possibly reflecting differences in risk perception, nicotine dependence, or access to alternative products.

These findings suggest that the public health advisory, together with heightened risk awareness following the EVALI outbreak, may have contributed to changes in e-cigarette use among adults. Following the Korean advisory, local media widely covered the government’s warning and convenience stores pulled e-cigarette products off shelves [24], increasing public attention to vaping-related risks. This combination of policy communication and risk awareness may have influenced user behavior. The fact that prevalence remained relatively low through 2023 suggests that the effect was not merely temporary but resulted in a lower baseline of e-cigarette use. However, because the post-2019 trend was flat rather than continuously declining, the advisory alone did not eliminate vaping, and some users either continued using or initiated e-cigarettes after the initial scare passed.

Our study adds to a body of literature examining how risk communication and regulatory actions impact tobacco use behavior. ITS analyses of the 2019 EVALI outbreak in the United States have similarly documented declines in e-cigarette sales and changes in tobacco product purchasing patterns during the outbreak period [25]. Previous studies have shown that the EVALI outbreak and subsequent media coverage heightened risk perceptions of e-cigarettes and were associated with declines in e-cigarette demand and sales [10, 16, 26]. Our findings from South Korea, a context outside the immediate U.S. outbreak but reacting to it, align with the notion that heightened risk awareness can reduce e-cigarette use. In Korea’s case, the government’s advisory likely amplified the risk message, and the magnitude of the observed decline is broadly consistent with the scale of behavior change expected during a nationwide vaping-related health scare.

The behavioral response observed in South Korea may also reflect broader sociocultural and institutional contexts. Previous research suggests that compliance with government recommendations in South Korea is often facilitated by relatively high institutional trust and norms of collective action. During the COVID-19 pandemic, trust in government and perceptions of institutional competence were strongly associated with adherence to preventive behaviors such as social distancing, handwashing, and quarantine compliance [27, 28]. Empirical studies also indicate that effective institutional responses increased public trust and cooperative behavioral responses among citizens [29, 30]. In addition, South Korea’s collectivist cultural orientation—often linked to Confucian traditions—emphasizes social harmony and group welfare [31], which may encourage compliance with public health recommendations even without strict regulatory enforcement. These sociocultural and institutional characteristics may partly explain why a non-binding advisory was followed by measurable behavioral change, while suggesting caution when generalizing these findings to other contexts.

It is also informative to contrast with countries that did not experience such a strong scare or intervention. In the United Kingdom, EVALI had minimal direct impact and public health authorities continued to affirm that regulated nicotine vaping products are less harmful than smoking. National surveys showed no significant drop in youth or adult vaping prevalence in 2019–2020, unlike in the United States, where youth vaping declined markedly following the EVALI outbreak and flavor bans [32]. This contrast suggests that Korea’s observed vaping decline may be linked to the advisory and surrounding circumstances rather than reflecting a global trend. Although vaping rates increased again in some countries after the EVALI and COVID-19 disruptions eased [33], Korea maintained a relatively lower level of use after 2019.

Crucially, our study found no sign that discouraging e-cigarette use drove people back to combustible cigarettes, a concern often raised in tobacco harm reduction debates [34, 35]. Critics have argued that restricting vaping could lead former users to relapse to smoking, potentially causing net public health harm if e-cigarettes were helping smokers avoid combustible products [36, 37]. However, we observed no increase in smoking prevalence following the 2019 advisory; instead, the long-term decline in smoking continued. This suggests that former vapers largely did not return to smoking, or that any relapse was too small to affect population trends. Evidence from other settings also indicates that vaping and smoking trends can move independently: for example, increases in e-cigarette use in the United States have coincided with faster declines in smoking prevalence [10], and Foxon et al. (2024) reported that higher e-cigarette prevalence was associated with lower adult smoking prevalence [38]. We also found no clear evidence of substitution toward HTPs. Although HTPs are sometimes considered alternatives to e-cigarettes [39, 40], and substitution has been observed in other contexts such as Japan [41], our results do not indicate that former vapers in South Korea switched to HTPs following the advisory. Previous studies likewise suggest that HTP uptake in Korea has largely occurred among dual users rather than as a direct replacement for e-cigarettes [18].

Our findings support the view that risk communication can function as a short-term public health tool, although sustaining its impact likely requires follow-up regulatory actions. In 2019, the South Korean MOHW issued a precautionary advisory against liquid-type e-cigarettes before any confirmed domestic fatalities, and this early intervention may have curtailed further uptake of vaping and encouraged some users to quit [13]. After the advisory, Korean authorities maintained the warning and proposed stricter regulations, although some measures faced delays, while manufacturers adapted by marketing non-nicotine or synthetic nicotine vape products that could circumvent certain rules [7]. Compared with formal legislation, advisories can be implemented rapidly and disseminated widely, but their effectiveness depends on voluntary compliance, public trust, and supportive regulatory frameworks. At the same time, precautionary advisories may generate controversy and require careful communication when scientific uncertainty remains about health risks [42, 43].

Our findings highlight the importance of a comprehensive regulatory framework to sustain reductions in e-cigarette use. While the 2019 advisory achieved a short-term decline, long-term impact will likely require policy measures such as flavor restrictions, ingredient disclosures, taxation, and extending smoke-free laws to include vaping consistent with WHO guidance on regulating electronic nicotine delivery systems [44]. Sustained public education also remains important, as clear communication about long-term harms—including dependence and respiratory and cardiovascular disease—may be particularly relevant for younger populations who may underestimate the risks [45]. Importantly, the absence of a post-advisory increase in smoking suggests that many vapers did not switch back to cigarettes, indicating an opportunity to combine advisories with cessation support, such as behavior counseling or nicotine replacement therapy, to facilitate complete tobacco abstinence. More broadly, these findings support Sustainable Development Goal (SDG) target 3.a and the WHO Framework Convention on Tobacco Control (FCTC), suggesting that timely precautionary risk communication can help curb emerging tobacco products without undermining progress in reducing cigarette smoking.

This study leveraged high-quality national survey data spanning over a decade, allowing us to discern secular trends and changes in tobacco use with greater confidence. By using an ITS design, a quasi-experimental approach for policy impact evaluation, we assessed changes associated with the 2019 advisory while accounting for baseline trends. Newey–West corrected standard errors were applied to address autocorrelation in annual aggregate data. We also conducted subgroup and sensitivity analyses to assess the robustness of the findings. This natural experiment provides real-world evidence on how risk perceptions and public health advisories may influence tobacco use behavior, and helps address gaps in the literature on e-cigarette policy impacts in Asian populations and older demographic groups.

Several limitations warrant caution. First, as an observational study, we cannot rule out the possibility that the association between advisory and changes in e-cigarette use was influenced by unmeasured contemporaneous factors, although we partially address this by showing no similar change in 2018 and by noting the specificity to e-cigarettes. Second, our outcome data are aggregated yearly prevalence estimates. Because the advisory occurred in October 2019, but the annual KNHANES 2019 data do not allow precise identification of when the decline occurred within late 2019 or early 2020, and short-term fluctuations may therefore be obscured. Third, the ITS analyses were based on aggregated annual prevalence estimates, which treat each time point as a single observation and do not fully account for within-timepoint heterogeneity or variation in sample size across survey years. Although weighted ITS approaches could improve precision, these methods were not feasible due to limited post-intervention observations and the absence of timepoint-specific variance measures [46]. Fourth, tobacco use was self-reported and therefore may be subject to under-reporting or misclassification; increased stigma following the advisory could have led some respondents to conceal vaping, although KNHANES is anonymous and has generally measured smoking reliably. Fifth, the relatively short follow-up period limits our ability to assess long-term substitution or relapse dynamics with emergence of new products such as synthetic nicotine devices. Finally, our ITS models assume linear trends and lack a contemporaneous control group because the advisory was nationwide. While cigarette use provides a partial comparison, we cannot fully rule out spillover effects or contextual influences, and the generalizability of the findings may be limited to settings with similar institutional and sociocultural conditions.

Our analysis highlights several avenues for future research. Individual-level longitudinal studies would complement these findings by examining whether e-cigarette users quit or switched after the advisory and whether such changes were sustained over time. Future research should also examine the impact of subsequent regulatory policies and the longer-term public health outcomes of the advisory. Evaluating measures such as potential flavor bans or product licensing systems may clarify whether stronger regulations reduce e-cigarette use or youth uptake. Comparative studies across countries in the Asia-Pacific region—where regulatory approaches range from outright bans to alternative regulatory frameworks—may further clarify the population health impacts of different regulatory regimes. Future studies should also assess whether reduction in e-cigarette use translates into measurable health outcomes, such as respiratory morbidity or smoking cessation patterns, and whether former e-cigarette users transition to complete nicotine abstinence.

## Conclusion

Our study found that the South Korean government’s 2019 advisory strongly warning against liquid e-cigarette use was associated with a significant reduction in e-cigarette prevalence at the population level, without evidence of adverse knock-on effects like increased smoking. This natural experiment underscores the power of timely public health interventions and communications in protecting population health. While the advisory achieved an immediate public health benefit by curtailing vaping, maintaining and capitalizing on this benefit will require comprehensive tobacco/nicotine control policies. As novel nicotine products proliferate globally, policymakers should proactively address their potential harms through a combination of surveillance, risk communication, and regulation. By continuing to adapt policies to emerging evidence, countries can ensure that progress in reducing tobacco-related disease is sustained and that the gains of the past decades are not undermined by a new generation of nicotine products.

## Supplementary Information

Supplementary Material 1. Figure S1. Trends in Heated Tobacco Product Use by Sex, 2019–2023. Table S1. Timeline of E-Cigarette Regulation in South Korea (2011 – 2025). Table S2. Annual sample sizes used in the interrupted time series (ITS) analyses (KNHANES, adults aged ≥19 years).

## Data Availability

The data supporting the findings of this study are publicly available from the Korea National Health and Nutrition Examination Survey (KNHANES) via the Korean Statistical Information Service (KOSIS) at [https://kosis.kr].
