# Supplementary material for: Differential impact of South Korea’s 2019 E-cigarette advisory on adult tobacco product use: an interrupted time series analysis by product type and subgroup
Source: BMC Public Health. 2026 Apr 24;26:1949. doi: 10.1186/s12889-026-27451-9 (PMC13289383; doi:10.1186/s12889-026-27451-9)
Supplement: Supplementary file 1 — Supplementary Material 1. Figure S1. Trends in Heated Tobacco Product Use by Sex, 2019–2023. Table S1. Timeline of E-Cigarette Regulation in South Korea (2011 – 2025). Table S2. Annual sample sizes used in the interrupted time series (ITS) analyses (KNHANES, adults aged ≥19 years). [file 12889_2026_27451_MOESM1_ESM.docx]

**Supplementary**

**Figure S1. Trends in Heated Tobacco Product Use by Sex, 2019–2023**

**Caption:** Annual prevalence (%) of current heated tobacco product use among adults aged ≥ 19 years in Korea. No abrupt increase in HTP use was observed following the October 2019 advisory on liquid-type e-cigarettes, suggesting minimal evidence of product substitution.

**Table S1. Timeline of E-Cigarette Regulation in South Korea (2011–2025)**

| **Year** | **Regulatory Measure and Description** | **Product Type** | **Key Features and Rationale** |
| --- | --- | --- | --- |
| 2011–2014 | Application of “tobacco” taxation and advertising restrictions to liquid-type e-cigarettes | Liquid-type | Classified as tobacco under the Tobacco Business Act; subject to the same rules as conventional cigarettes (advertising limits, retail licensing, smoke-free zones). |
| 2015 | Amendment to the National Health Promotion Act: inclusion of e-cigarettes in public indoor smoking bans | All e-cigarettes | Expanded indoor smoke-free zones; introduced health warning labels on e-cigarette packages. |
| Jun 2017 | Market introduction of heated tobacco products (IQOS, glo) | Heated tobacco | Industry marketed as “reduced-risk”; MFDS analysis found tar and nicotine levels comparable to conventional cigarettes. |
| 2018 | Ministry of Education strengthened school-based anti-vaping guidelines | All e-cigarettes | Added e-cigarettes to school smoking-prevention curricula; reinforced youth education. |
| **Oct 2019** | **MOHW issued public advisory to refrain from using liquid-type e-cigarettes (Press Release, Oct 23 2019)** | **Liquid-type** | **Response to U.S. EVALI outbreak (1,479 cases, 33 deaths); advised public to stop use pending safety verification; sales plummeted.** |
| Nov 2019 | MFDS testing of domestic liquid-type products for THC and vitamin E acetate | Liquid-type | No THC or vitamin E acetate detected in Korean products; confirmed different composition from U.S. cases. |
| Jan 2020 | MOHW announced roadmap for stronger tobacco and e-cigarette control | All e-cigarettes | Proposed product approval system, flavor restrictions, and tax increase; some measures remain under review. |
| 2020–2022 | Expansion of graphic warnings to heated tobacco products | Heated tobacco | Unified warning standards across all tobacco types; aligned risk communication with conventional cigarettes. |
| May 2023 | Proposed amendment to the Tobacco Business Act (product classification and flavor ban) | All types | Introduced differential taxation by product category and product authorization system (pending parliamentary approval). |
| From Jan 2024 | Strengthened enforcement against illegal sales and use of e-cigarettes in schools | Youth-targeted products | Education offices expanded inspections and prevention campaigns; developed digital content for Gen Z. |
| Jun 2025 | Administrative pre-announcement of the Regulation on Harmful Constituents in Tobacco Products (Public Notice No. 2025 − 250) | All tobacco products including e-cigarettes | Issued by MFDS following the Tobacco Hazard Management Act (Enacted Oct 31 2023; Effective Nov 1 2025). Designates 44 harmful constituents for cigarettes and heated tobacco, and 20 for liquid-type e-cigarettes; establishes o |

**Table S2.** **Annual sample sizes used in the interrupted time series (ITS) analyses (KNHANES, adults aged ≥ 19 years)**

| **Year** | **Cigarette smoking (N)** | **Liquid-type e-cigarette use (N)** | **Heated tobacco product use (N)** |
| --- | --- | --- | --- |
| 2007 | 5,338 | – | – |
| 2008 | 5,192 | – | – |
| 2009 | 5,405 | – | – |
| 2010 | 6,015 | – | – |
| 2011 | 6,115 | – | – |
| 2012 | 6,183 | – | – |
| 2013 | 5,338 | 5,338 | – |
| 2014 | 5,192 | 5,190 | – |
| 2015 | 5,405 | 5,405 | – |
| 2016 | 6,015 | 6,015 | – |
| 2017 | 6,115 | 6,114 | – |
| 2018 | 6,183 | 6,183 | – |
| 2019 | 6,190 | 6,190 | 6,190 |
| 2020 | 5,858 | 5,858 | 5,858 |
| 2021 | 5,630 | 5,630 | 5,630 |
| 2022 | 5,254 | 5,254 | 5,254 |
| 2023 | 5,763 | 5,763 | 5,763 |

**Note.** Values represent the annual number of survey respondents aged ≥ 19 years contributing to each outcome, as reported in publicly available KNHANES summary statistics accessed via the Korean Statistical Information Service (KOSIS). Liquid-type e-cigarette use was first measured in 2013, and heated tobacco product use was first measured in 2019. All analyses were conducted using aggregated prevalence estimates derived from repeated cross-sectional survey data. A dash (–) indicates that the outcome was not measured in that survey year.
